# Susceptibility of Human Plasma N-glycome to Low-Calorie and Different Weight-Maintenance Diets

**DOI:** 10.3390/ijms232415772

**Published:** 2022-12-12

**Authors:** Helena Deriš, Petra Tominac, Frano Vučković, Arne Astrup, Ellen E. Blaak, Gordan Lauc, Ivan Gudelj

**Affiliations:** 1 Genos Glycoscience Research Laboratory, 10000 Zagreb, Croatia; 2 Department of Nutrition, Exercise and Sports, University of Copenhagen, DK 2200 Copenhagen, Denmark; 3 Department of Human Biology, NUTRIM, School for Nutrition and Translational Research in Metabolism, Maastricht University, 6200 MD Maastricht, The Netherlands; 4 Faculty of Pharmacy and Biochemistry, University of Zagreb, 10000 Zagreb, Croatia; 5 Department of Biotechnology, University of Rijeka, 51000 Rijeka, Croatia

**Keywords:** plasma N-glycans, weight loss, low-calorie diet, obesity

## Abstract

Aberrant plasma protein glycosylation is associated with a wide range of diseases, including diabetes, cardiovascular, and immunological disorders. To investigate plasma protein glycosylation alterations due to weight loss and successive weight-maintenance diets, 1850 glycomes from participants of the Diogenes study were analyzed using Ultra-High-Performance Liquid Chromatography (UHPLC). The Diogenes study is a large dietary intervention study in which participants were subjected to a low-calorie diet (LCD) followed by one of five different weight-maintenance diets in a period of 6 months. The most notable alterations of the plasma glycome were 8 weeks after the subjects engaged in the LCD; a significant increase in low-branched glycan structures, accompanied by a decrease in high-branched glycan structures. After the LCD period, there was also a significant rise in N-glycan structures with antennary fucose. Interestingly, we did not observe significant changes between different diets, and almost all effects we observed immediately after the LCD period were annulled during the weight-maintenance diets period.

## 1. Introduction

According to the World Health Organization, in 2016 almost 40% of adults were overweight (BMI ≥ 25 kg/m^2^) and around 13% of the world’s adult population were obese (BMI ≥ 30 kg/m^2^), and these numbers only seem to be increasing worldwide [1]. Being overweight or obese poses a serious threat to common health since it adversely affects nearly all physiological functions of the body. Global BMI Mortality Collaboration analyzed data from 10.6 million adults enrolled in 239 prospective studies from thirty-two countries worldwide and showed that mortality was lowest in the BMI range from 20.0 kg/m^2^ to 25.0 kg/m^2^. Both overweight and obesity are associated with increased all-cause mortality [2]. Having a higher BMI augments the risk of developing a variety of diseases, cardiovascular diseases [3,4], diabetes mellitus [5,6], and several types of cancer [7], as well as depression [8]. Thus, weight loss interventions and healthy weight maintenance are important for the prevention and treatment of overweight/obesity to abate the risk of developing BMI-related health complications.

Despite weight loss in overweight or obese individuals was shown to reduce the risk of all-cause mortality [9], risk of CVD complications [10], and incidence of type 2 diabetes [11] even in a 10-year follow-up [12], one study suggested that weight loss differently affects people with T2D and those differences were BMI-dependent [13]. The amount of weight loss, as well as the timing of weight loss in the course of the disease, may differentially affect the risk of CVD and mortality [13]. Thus, studies of the weight loss impact on cancer, T2D, and CVD morbidity and mortality may have inconclusive or contradictory results. The reason behind it might be due to common weight regain that follows the initial weight loss, which usually happens in the first year after T2D or CVD diagnosis [13]. Once the targeted weight loss is achieved, maintaining that weight is often even harder than the weight loss itself. Therefore, maintaining the preferable weight is also important as it was shown that major weight variability was associated with an increased risk of mortality and cardiovascular outcomes in overweight and obese patients with diabetes [14].

Long-term weight maintenance is often hard to achieve; around 20% of people in the general population are successful at long-term weight maintenance [15], with long-term weight maintenance being defined as “intentionally loosing at least 10% of one’s body weight and keeping it off at least one year” [16]. Long-term weight control is challenging because of the interactions between our biology, psychology, and the obesogenic environment [17]. The most common weight loss strategies have often been used for weight management as well, and rely primarily on maintaining behavioral changes that involve more internal forms of motivation, active self-regulation, and constraints including resources and environmental factors [18]. Very few people consistently maintain that kind of lifestyle throughout their adulthood. Instead, most people go through repeated cycles of ad libitum dieting and calorie restrictive dieting, the so-called yo-yo diet, with very well-documented adverse effects [19,20,21]. For these reasons, more and more research into weight maintenance and related cardiometabolic health tend to investigate the individual impact of diet composition by studying different molecular processes such as post-translational modifications (PTMs). One of the most common PTMs is N-glycosylation. N-glycosylation is a co- and post-translational modification of proteins in which carbohydrate moieties are attached to an asparagine residue of a polypeptide backbone. It is considered one of the most diverse modifications as there are numerous possible N-glycan structures, despite glycan biosynthesis not being template-driven [22]. It is dependent on expression, activity, and turnover of many enzymes, as well as the availability of sugar donors, all of which results in a multitude of possible N-glycan structures that are found on different glycoproteins [23]. Plasma N-glycans are rather stable in healthy individuals over time [24,25], but they reflect physiological [26,27], pathological [28,29,30,31,32], or lifestyle [26] changes within a person, since glycosylation influences stability and physical properties of proteins. Protein glycosylation is involved in various biological processes such as receptor interaction, immune response, and protein secretion and transport [33]. Studying plasma proteins’ glycosylation may offer a wealth of information, although sometimes it may be hard to tell whether certain N-glycan changes are the result of the protein-specific changes in glycan composition or alterations in abundances of individual proteins with characteristic glycans [33]. Overall levels of the sialyl Lewis X epitope of plasma proteins were found to be increased in chronic and acute inflammation [34], increased galactosylation, sialylation, and branching were associated with higher risk of type 2 diabetes [35] and insulin resistance [36]. Levels of plasma protein antennary fucosylation are being investigated as a potential biomarker for differentiation of maturity onset diabetes of the young (MODY) [37,38]. Decreased sialylation and galactosylation of IgG have been reported in type 2 diabetes and hypertension, while increased IgG core fucosylation has also been associated with hypertension. On the other hand, the occurrence of bisecting IgG glycan structures was decreased in hypertension and increased in T2D [39]. One study showed that an increase in the non-galactosylated and a decrease in digalactosylated plasma N-glycans as well as an increase in sialylation of biantennary structures are associated with increased body fat and blood pressure [26]. Moreover, when looking at immunoglobulin G N-glycans, a negative association between BMI and the level of neutral glycans with two terminal galactoses was found suggesting that BMI can be responsible for up to 3.2% of variation in this glycan feature [40]. Therefore, it comes as no surprise that protein N-glycosylation changes reflect changes in diet, weight, and BMI [41,42].

Given the importance of glycosylation and its susceptibility to reflect (patho)physiological changes in an individual, we conducted the analysis of 1850 total plasma proteins’ N-glycomes from participants of the Diogenes study. The Diogenes study is a randomized, controlled dietary intervention study conducted in eight European centers: Maastricht (Netherlands), Copenhagen (Denmark), Cambridge (UK), Heraklion (Greece), Potsdam (Germany), Pamplona (Spain), Sofia (Bulgaria), and Prague (the Czech Republic). The study participants were subjected to an eight-week low-calorie diet (LCD) followed by one of five weight-maintenance diets (low protein (LP)/low glycemic index (LGI), low protein (LP)/high glycemic index (HGI), high protein (HP)/LGI, HP/HGI and control) in a period of six months when the participants were at risk of regaining the formerly lost weight.

## 2. Results

N-glycome composition of subjects’ plasma samples was determined by UHPLC analysis of glycans labelled with 2-aminobenzamide as described in the Section 4. Statistical analysis was performed on 16 glycan-derived traits calculated from 39 directly measured glycan structures, corresponding to 39 glycan peaks obtained by UHPLC analysis (Figure S1).

**Figure 1:**
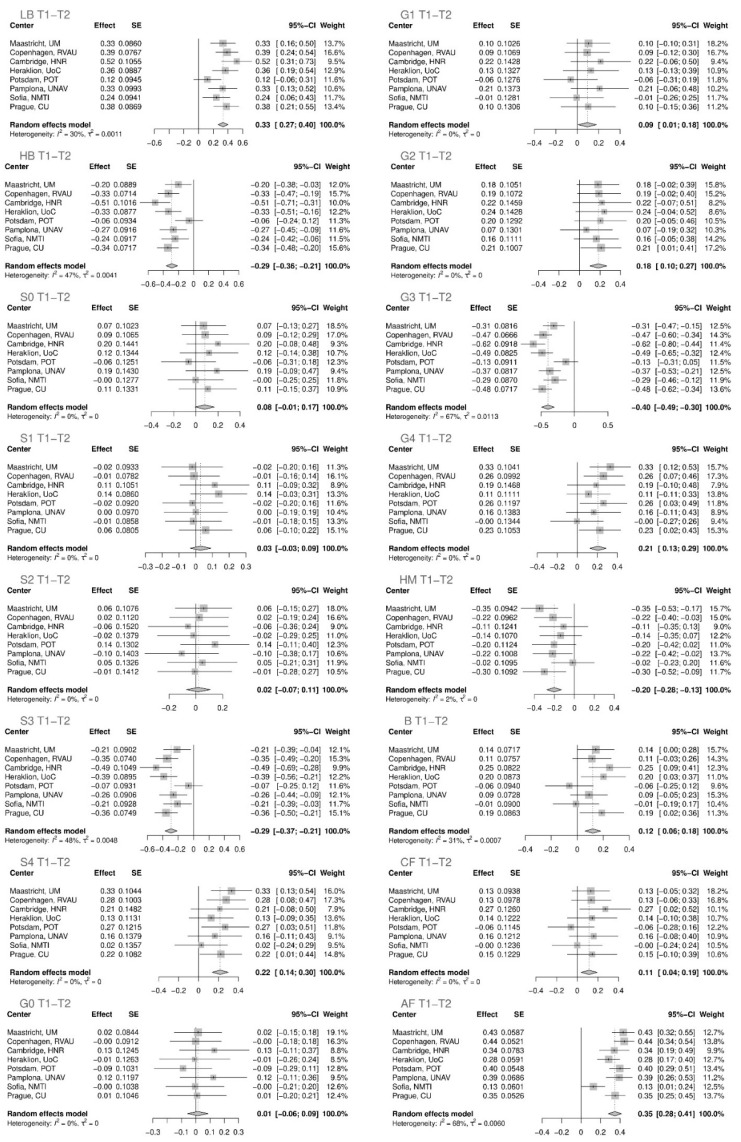
The effect of a low-calorie diet on plasma N-glycome composition in the first time period of eight weeks (T1–T2). Changes in plasma protein glycome composition after performing meta-analysis for all the centers are shown. SE—standard error; 95% CI –95% confidence interval; T1—time point 1; T2—time point 2; T3—time point 3; LB—total low branching glycans; HB—total high branching glycans; S0—total neutral glycans; S1—total monosialylated glycans; S2—total disialylated glycan; S3—total trisialylated glycans; S4—total tetrasialylated glycans; G0—total agalactosylated glycans; G1—total monogalactosylated glycans; G2—total digalactosylated glycans; G3—total trigalactosylated glycans; G4—total tetragalactosylated glycans; HM—total high-mannose glycans; B—total glycans with bisecting GlcNAc; CF—total glycans with core fucose; AF—total glycans with antennary fucose.

**Figure 2:**
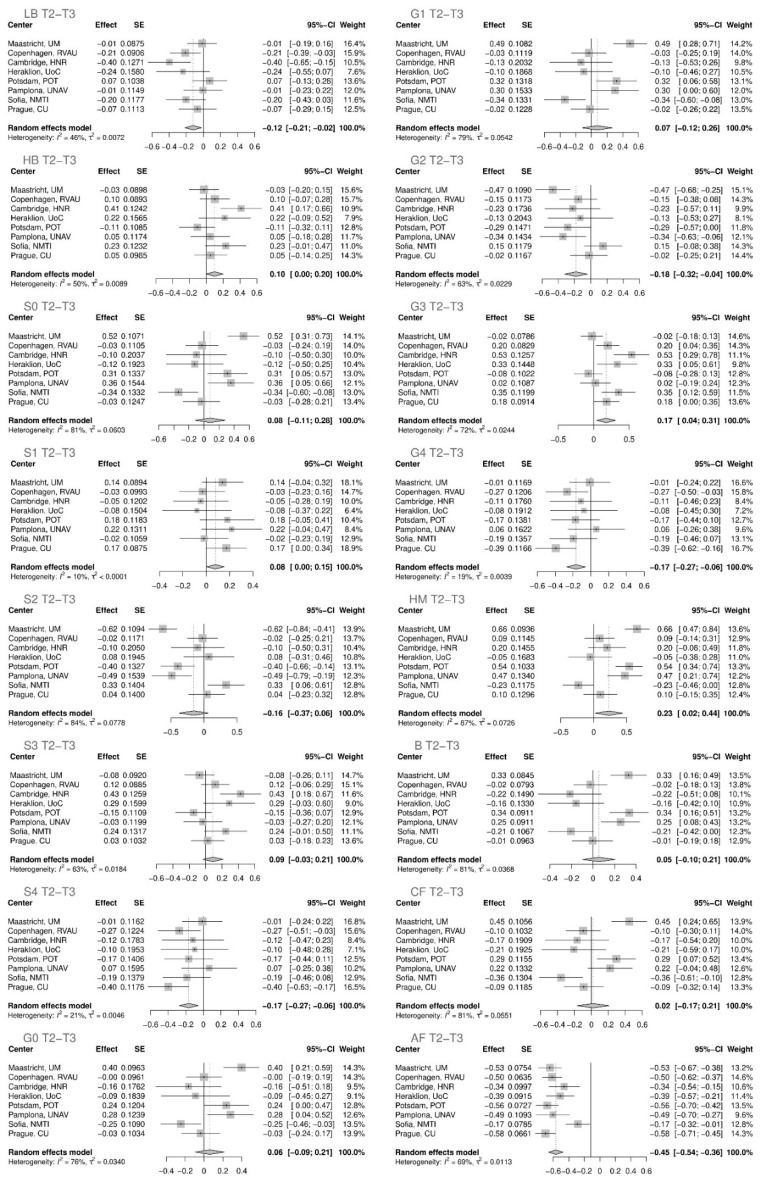
The effect of different weight-maintenance diets on plasma N-glycome in the second time period of 26 weeks (T2–T3). Changes in plasma protein glycome composition after performing meta-analysis for all the centers are shown. SE—standard error; 95% CI –95% confidence interval; T1—time point 1; T2—time point 2; T3—time point 3; LB—total low branching glycans; HB—total high branching glycans; S0—total neutral glycans; S1—total monosialylated glycans; S2—total disialylated glycans; S3—total trisialylated glycans; S4—total tetrasialylated glycans; G0—total agalactosylated glycans; G1—total monogalactosylated glycans; G2—total digalactosylated glycans; G3—total trigalactosylated glycans; G4—total tetragalactosylated glycans; HM—total high-mannose glycans; B—total glycans with bisecting GlcNAc; CF—total glycans with core fucose; AF—total glycans with antennary fucose.

The statistical analysis was first performed on the rank transformed glycan variables for each Diogenes research center independently (Figure S2). Subsequently, meta-analysis was performed for all centers of the Diogenes study for the first period, T1–T2 (Figure 1), and the second period of the study, T2–T3 (Figure 2).

**Figure 3:**
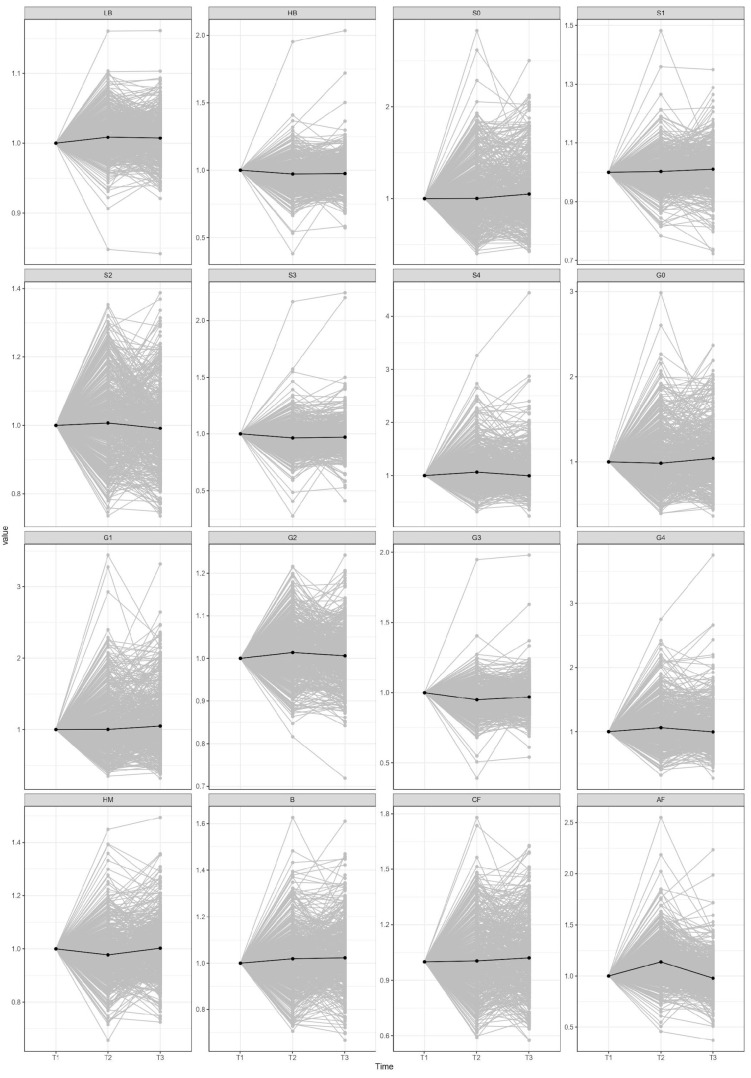
Changes in plasma N-glycome composition between different time-periods. Presented glycome changes are normalized to the first point. T1—time point 1; T2—time point 2; T3—time point 3; LB—total low branching glycans; HB—total high branching glycans; S0—total neutral glycans; S1—total monosialylated glycans; S2—total disialylated glycans; S3—total trisialylated glycans; S4—total tetrasialylated glycans; G0—total agalactosylated glycans; G1—total monogalactosylated glycans; G2—total digalactosylated glycans; G3—total trigalactosylated glycans; G4—total tetragalactosylated glycans; HM—total high-mannose glycans; B—total glycans with bisecting GlcNAc; CF—total glycans with core fucose; AF—total glycans with antennary fucose.

Eleven out of sixteen derived glycan traits showed statistically significant variations in their levels in the first time period (Figure 3, Table 1) while in the second time period only six of them remained statistically significant (Figure 2, Table 2) after adjustment for multiple testing (adjusted *p* value < 0.05).

In the first time period (T1–T2), when subjects were on an 8-week LCD (Table 1, Figure 1), the most prominent changes in specific glycan structure levels were an increase in glycan structures with the antennary fucose (adjusted *p* value < 4.93 × 10^−22^) and an increase in low-branched glycan structures (adjusted *p* value < 2.26 × 10^−21^). The letter one is followed by a concomitant decrease in highly branched structures (adjusted *p* value < 2.32 × 10^−13^), such as trigalactosylated (adjusted *p* value < 4.10 × 10^−16^) and trisialylated (adjusted *p* value < 1.01 × 10^−12^) glycan structures. Tetrasialylated (adjusted *p* value < 7.54 × 10^−7^), tetragalactosylated (adjusted *p* value < 1.90 × 10^−6^), as well as digalactosylated (adjusted *p* value < 3.29 × 10^−5^) glycan structures showed the statistically significant increase after the LCD. Moreover, high-mannose glycans (adjusted *p* value < 2.22 × 10^−7^) decreased, while bisecting glycans (adjusted *p* value < 0.00024) and core-fucosylated glycans (adjusted *p* value < 0.00959) increased their levels.

In the second time period (T2–T3), when subjects were going through one of the five weight-maintenance diets (Table 2, Figure 2), undoubtedly the most striking change was a decrease in glycan structures with the antennary fucose (adjusted *p* value < 1.91 × 10^−20^). In addition, tetrasialylated structures (adjusted *p* value < 0.00661), tetragalactosylated structures (adjusted *p* value < 0.00580), and digalactosylated glycan structures (adjusted *p* value < 0.02353) and low-branched glycans (adjusted *p* value < 0.03186) decreased, while trigalactosylated (adjusted *p* value < 0.01873) and high-mannose glycans (adjusted *p* value < 0.04994) increased.

When comparing the effect of different weight-maintenance diets on glycome composition during the second time period (T2–T3) we did not see any significant differences between them (Supplementary Figure S3).

## 3. Discussion

During the 8-week period on the LCD, most people lost > 8% of their initial body weight [43], subsequentially reducing their average concentrations of high-sensitivity C-reactive protein (CRP) [44]. Obesity causes a state of a chronic low-grade systemic inflammation [45], with elevated levels of certain circulating proinflammatory adipokines, such as TNF-α, IL−6, leptin, plasminogen activator inhibitor-1 (PAI-1), angiotensinogen, and CRP [46], as well as lower levels of adiponectin [47]. Moreover, during the chronic inflammation, other APPs mainly secreted by hepatocytes, such as α1-acid glycoprotein (AGP) [48,49], α1-antitrypsin (A1AT) [50], α1-antichymotrypsin (AACT) [50], and haptoglobin (HPT) [50,51], exhibit not only altered serum concentrations [52,53] in response to proinflammatory cytokines [54], but also altered glycosylation [28,34,55].

The most prominent changes in the T1-T2 period of the Diogenes study were the increase in glycan structures with antennary fucose and low-branched glycan structures. The increase in low-branched, mono- and biantennary, glycan structures and accompanied decrease in high-branched glycan structures may primarily be contributed to the decreased levels of triantennary structures, trigalactosylated and trisialylated, as well as the increase in digalactosylated glycans. Other low-branched glycosylation features, such as monogalactosylated, disialylated, and monosialylated glycan structures exhibit the positive trend in their levels likewise, although not statistically significant. Elevated levels of complex high-branched and concomitant lower levels of low-branched glycan structures were found in many different chronic diseases, such as chronic obstructive pulmonary disease [56], chronic low back pain [57], T2D [35], and ovarian cancer [58], and are commonly related to chronic inflammation. Moreover, the same patterns of glycan branching as a response to a disease have been related to the specific serum proteins. High branching has been associated with glycosylation changes in some major APPs; transferrin glycans showed increased branching in rheumatoid arthritis [59] and ulcerative colitis [60], HPT glycans exhibited higher branching in pancreatic, hepatic, ovarian, and prostate cancer [61], A1AT glycans in hepatocellular carcinoma [62], while AACT showed higher branching in septic patients [63]. Therefore, decreased high branching and increased low branching of plasma proteins confirm that the LCD and subsequent weight loss correlate with the mitigation of chronic inflammation.

Contrary to the overall high-branched glycan structures, tetragalactosylated and tetrasialylated structures increased during the first time period. Tetra-antennary glycan structures are considered to primarily originate from AGP [64,65]. Increased levels of all AGP highly branched glycan structures (tri- and tetra-antennary alike) have previously been associated with different chronic inflammatory conditions [65]. However, it seems that AGP levels are increased during calorie restriction which, at least partially explains the observed increase in tetragalactosylated and tetrasialylated structures [66,67]. Moreover, enhanced levels of antennary fucose on glycan structures are also a consequence of the increased AGP levels since most of the structures with α1,3 linked fucose on tri-, and tetra-antennary sialylated glycan predominantly originate from AGP. Changes in glycan structures originating from the AGP in response to LCD may not be solely due to the changes in AGP concentration. AGP may regulate food intake and energy homeostasis in response to nutrition status through the leptin receptor [68] and it may in fact protect adipose tissue from inflammation and metabolic dysfunction in mice by suppressing proinflammatory gene expression and pathways such as NF-κB and mitogen-activated protein kinase signaling and reactive oxygen species generation [69]. AGP glycans account for 42% of its molecular weight [70]; however, their exact role in these processes is yet to be elucidated.

Other glycosylation features that changed significantly after the 8-week LCD diet were decrease in high-mannose-type glycans and rise in bisecting and core fucosylation. High-mannose (M5–M9) glycans in human plasma proteins are predominantly derived from the apolipoprotein B-100 (ApoB) and immunoglobulin M (IgM) [33]. ApoB transports most of the plasma cholesterol and has a major role in the assembly of atherogenic low- and very low-density lipoproteins [33], and it has been shown that its concentration decreases during calorie restriction [71]. Yet, due to the analytical method used for plasma N-glycan analysis, it is hard to say whether the reduction in total plasma protein high-mannose glycans after LCD is solely caused by the change in ApoB concentrations or by the changes in ApoB glycosylation, or both. Core fucosylation also shows a slight increase after the LCD; even though the majority of core fucosylated glycans originate from the IgG, we recently showed that IgG core fucosylation insignificantly decreases after LCD [72] and therefore observed changes cannot be attributed to this plasma protein. Moreover, IgG plasma core fucosylated N-glycan structures may originate from other immunoglobulins such as IgA and IgM as well as from apolipoproteins D and E, which means alterations in concentrations and/or glycosylation are the probable source of increased core fucosylation.

Plasma protein glycosylation alterations after the LCD-induced weight loss mainly reflect the anti-inflammatory glycosylation pattern [73,74], which is in line with the Diogenes study IgG glycosylation analysis [72].

Observed glycosylation alterations in the first time period were largely nullified after the six months on different weight maintenance regimes (Figure 3), which suggests that mainly the weight loss itself steers the glycosylation changes in plasma proteins. Moreover, there was no significant difference in glycosylation patterns between different types of weight-maintenance diets. Even though it was published that a diet rich in refined grain intake was associated with increased total fucosylation and reduced total sialylation [41] of serum proteins compared with dietary intake of vegetables and dairy, it is necessary to conduct further research to gain a more meticulous insight into dietary influence on plasma/serum protein glycosylation.

## 4. Material Methods

### 4.1. Subjects

Plasma glycoproteins’ glycome composition was determined by analyzing 1850 blood plasma samples collected at eight centers of Diogenes study, previously described in detail [43,75,76]. Briefly, 938 overweight or obese adults, with the mean age of 41 years and mean BMI of 34 kg m^−2^, entered the first phase of the study, the low-calorie diet phase. Participants (*n* = 773) who achieved the targeted weight loss (≥8% of their baseline weight) during the 8-week LCD period were randomly assigned to one of five maintenance diets for next six months. Randomization was performed using a simple block randomization method with stratification. Blood was collected at three different time points; at the beginning of the LCD intervention (time point 1, T1), after eight weeks on the LCD diet (time point 2, T2), and after six months on a weight-maintenance diet (time point 3, T3). More information about the Diogenes participants included in the plasma protein glycosylation analysis can be found in our recently published study [72].

### 4.2. Sample Preparation

Sample preparation and glycan analysis were carried out using the high-throughput method. Block randomization was used to determine the position of samples in 26 96-well plates. There were approximately 70 samples in each 96-well plate along with five randomly selected technical replicate samples from the same plate and five from other plates. In addition to the sample replicates, four internal plasma standards were included in each plate to maintain quality control and allow batch correction to be performed later.

The samples were prepared as previously described [77]. Briefly, 10 μL of each plasma sample was pipetted into the sample collection plates (Waters, Milford, CT, USA) and 20 µL of the detergent, 2% (*w*/*v*) sodium dodecyl sulfate (Invitrogen, Carlsbad, NM, USA) was added to all samples. Samples were incubated at 65 °C for 10 min to successfully denature plasma proteins. A total of 10 μL of 4% (*v*/*v*) Igepal CA-630 (Sigma-Aldrich, St. Louis, MO, USA) was added to the samples to prevent undesired denaturation of the enzyme PNGase F (peptide-N-(N-acetyl-glucosaminyl)-asparaginamidase) F; Promega, Madison, USA) which in the next step was added as a mixture with 5x phosphate buffer saline (5x PBS, prepared in-house) in a total volume of 10 μL per sample. Plasma protein deglycosylation was performed at 37 °C for 18 h. After deglycosylation was complete, the samples were fluorescently labeled with 25 μL of a mixture of 2-aminobenzamide (2-AB; Sigma-Aldrich) and 30% glacial acetic acid (Merck, Germany) in dimethyl sulfoxide (Sigma-Aldrich), and incubated for 2 h at 65 °C. To remove the excess of reagents, 2-AB-labeled N-glycans were purified by hydrophilic liquid chromatography solid-phase extraction (HILIC-SPE) using 0.2 μm wwPTFE 96-well membrane filter plates (Pall, New York, NY, USA). The samples were continuously washed with freshly prepared 96% acetonitrile (ACN) and the purified labeled glycans were eluted with 2 × 90 µL of ultrapure water and stored at −20 °C until further use.

### 4.3. Hydrophilic Interaction Chromatography—Ultra-High-Performance Liquid Chromatography with Fluorescence Detection (HILIC-UHPLC-FLR) N-glycan Analysis

Analysis of 2-AB-labeled plasma protein N-glycans was performed on three Waters Acquity UPLC H-class instruments monitored by Waters Empower 3 software and consisting of a quaternary solvent manager, a sample manager and a fluorescence detector set with excitation and emission wavelengths of 330 and 420 nm, respectively. Labeled N-glycans were separated using Waters UPLC Glycan bridged ethylene hybrid (BEH) Amide chromatographic columns (130 Å, 1.7 µm BEH particles, 2.1 × 10 mm) with 100 mmol/L freshly prepared ammonium formate, pH 4.4 as solvent A, and 100% LC-MS grade ACN (Honeywell, Charlotte, NC, USA) as solvent B. The separation method included a linear gradient of 70–53% acetonitrile (*v*/*v*) at a flow rate of 0.561 mL mL/min over 25 min in a 32.5 min analytical run with the injection volume of 20 µL. The system was calibrated with an external standard of hydrolyzed and 2-AB-labeled glucose oligomers, from which the individual glycan retention times were translated into glucose units (GUs). Chromatograms acquired in the analysis were processed using the automated integration method and separated into 39 glycan peaks (GP31–GP39). Glycan peaks were analyzed by their elution positions and then measured in glucose units, which were compared with reference values found in the GlycoStore database (available at: https://glycostore.org/, accessed on 15 January 2022) for structure assignment. A detailed interpretation of the glycan structures corresponding to each glycan peak is presented in Supplementary Figure S1. total area normalized (%Area) values were obtained for each peak to enable relative quantification of plasma N-glycans.

### 4.4. Data Analysis

#### 4.4.1. Normalization and Batch Correction

Normalization and batch correction were performed on UHPLC glycan data to eliminate experimental variation in measurements. To remove experimental noise and make the glycan peak measurements comparable across samples regardless of their absolute intensities, a total area normalization was performed. The peak area of each of the 39 glycan structures obtained directly was divided by the total area of the corresponding chromatogram and multiplied by 100, with each peak being expressed as a percentage of the total integrated area. Before batch correction, normalized glycan measurements were log-transformed due to the right skewness of their distributions and the multiplicative nature of batch effects. Batch correction was performed on logarithmically transformed measurements using the ComBat method (R package sva) [78], where the technical source of variation, the number of sample plates, was modeled as a batch covariate. This was performed for each glycan peak. Estimated batch effects were subtracted from logarithmically transformed measurements to provide measurement correction for experimental noise.

#### 4.4.2. Derived Traits

Sixteen derived traits were calculated from 39 glycan structures directly obtained by UHPLC analysis. These derived glycan traits represent a share of structurally similar glycan groups with joint biosynthetic pathways. Total plasma protein-derived glycan traits were calculated as the ratios of glycan peaks (GP1-GP39) with the same structural characteristics in a total plasma protein glycome: total low branching glycans (mono- and biantennary glycans), LB = (GP1 + GP2 + GP3 + GP4 + GP5 + GP6 + GP8 + GP9 + GP10 + GP11 + 0.5 × GP12 + GP13 + GP14 + GP15 + GP16 + GP17 + GP18 + GP20 + GP21 + GP22 + GP23)/SUM (GP1-GP39) × 100; total high branching glycans (tri- and tetraantennary glycans), HB = (GP24 + GP25 + GP26 + GP27 + GP28 + GP29 + GP30 + GP31 + GP32 + GP33 + GP34 + GP35 + GP36 + GP37 + GP38 + GP39)/SUM (GP1 − GP39) × 100; total agalactosylated glycans, G0 = (GP1 + GP2)/SUM (GP1 − GP39) × 100; total monogalactosylated glycans, G1 = (GP3 + GP4 + GP5 + GP6 + GP13)/SUM (GP1 − GP39) × 100; total digalactosylated glycans, G2 = GP8 + GP9 + GP10 + GP11 + 0.5xGP12 + GP14 + GP15 + GP16 + GP17 + GP18 + GP20 + GP21 + GP22 + GP23)/SUM (GP1 − GP39) × 100; total trigalactosylated glycans, G3 = (GP24 + GP25 + GP26 + GP27 + GP28 + GP29 + GP30 + GP31 + GP32 + GP33 + GP34+ GP35)/SUM (GP1 − GP39) × 100; total tetragalactosylated glycans, G4 = (GP36 + GP37 + GP38 + GP39)/SUM (GP1 − GP39) × 100; total neutral glycans, S0 = (GP1 + GP2 + GP3 + GP4 + GP5 + GP6 + GP8 + GP9 + GP10 + GP11)/SUM (GP1 − GP39) × 100; total monosialylated glycans, S1 = (0.5xGP12 + GP13 + GP14 + GP15 + GP16 + GP17)/SUM (GP1 − GP39) × 100; total disialylated glycans, S2 = (GP18 + GP20 + GP21 + GP22 + GP23 + GP24 + GP25 + GP26 + GP27)/SUM (GP1 − GP39) × 100; total trisialylated glycans, S3 = (GP28 + GP29 + GP30 + GP31 + GP32 + GP33 + GP34 + GP35 + GP36)/SUM (GP1 − GP39) × 100; total tetrasialylated glycans, S4 = (GP37 + GP38 + GP39)/SUM (GP1 − GP39) × 100; total glycans with bisecting GlcNAc, B = (GP2 + GP3 + GP6 + GP9 + GP11 + GP15 + GP17 + GP23)/SUM (GP1 − GP39) × 100; total glycans with antennary fucose, AF = (GP27 + GP33 + GP35 + GP39)/SUM (GP1 − GP39) × 100; total glycans with core fucose, CF = (GP1 + GP2 + GP4 + GP5 + GP6 + GP10 + GP11 + GP13 + GP16 + GP17 + GP22 + GP23 + GP31 + GP34 + GP35)/SUM (GP1 − GP39) × 100; total high-mannose glycans, HM = (GP7 + 0.5 × GP12 + GP19)/SUM (GP1 − GP39) × 100.

#### 4.4.3. Longitudinal Analysis

Longitudinal analysis of patient samples during their observation period was performed by implementing a linear mixed effects model R-package lme4 [79], where glycan measurement was the dependent variable, time was modeled as a fixed effect, the individual ID was included in the model as a random intercept, with age, sex, and BMI included as additional covariates. The analyses were initially performed separately for each center and then combined using a random effects meta-analysis approach (R-package meta, metagen; method = “ML”) [80]. Prior to the analyses, all glycan variables were transformed to a standard normal distribution (mean = 0, sd = 1) by the inverse transformation from ranks to normality (R package “GenABEL”, function rntransform) [81]. The use of rank-transformed variables in analyses makes estimated effects of different glycans in different centers comparable because transformed glycan variables have the same standardized variance. The false detection rate was controlled using the Benjamini–Hochberg method (function p.adjust(method = BH)). The data were analyzed and visualized using the R programming language (version 3.0.1).

## Figures and Tables

**Table 1:** Changes in plasma N-glycome composition in the first time period (T1–T2) after meta-analysis. Eleven out of sixteen derived glycan traits showed statistically significant changes in their levels after subjects spent 8 weeks on the low-calorie diet (adjusted *p*-value < 0.05). AF—total glycans with antennary fucose; LB—total low branching glycans; HB—total high branching glycans; S0—total neutral glycans; S1—total monosialylated glycans; S2—total disialylated glycans; S3—total trisialylated glycans; S4—total tetrasialylated glycans; G0—total agalactosylated glycans; G1—total monogalactosylated glycans; G2—total digalactosylated glycans; G3—total trigalactosylated glycans; G4—total tetragalactosylated glycans; HM—total high-mannose glycans; B—total glycans with bisecting GlcNAc; CF—total glycans with core fucose.

	T1–T2			
Glycan	Effect	Standard Error	*p* Value	Adjusted *p* Value
AF total	0.34665	0.03467	1.54 × 10^−23^	4.93 × 10^−22^
LB total	0.33330	0.03409	1.41 × 10^−22^	2.26 × 10^−21^
G3 total	−0.39682	0.04733	5.12 × 10^−17^	4.10 × 10^−16^
HB total	−0.28782	0.03800	3.63 × 10^−14^	2.32 × 10^−13^
S3 total	−0.29167	0.03965	1.89 × 10^−13^	1.01 × 10^−12^
HM total	−0.20367	0.03733	4.86 × 10^−8^	2.22 × 10^−7^
S4 total	0.21731	0.04171	1.88 × 10^−7^	7.54 × 10^−7^
G4 total	0.20656	0.04120	5.34 × 10^−7^	1.90 × 10^−6^
G2 total	0.18442	0.04181	1.03 × 10^−5^	3.29 × 10^−5^
B total	0.11955	0.03034	8.13 × 10^−5^	2.36 × 10^−4^
CF total	0.11460	0.04003	0.00419	0.00959
G1 total	0.09387	0.04375	0.03190	0.05373
S0 total	0.08067	0.04394	0.06639	0.09657
S1 total	0.02901	0.03133	0.35451	0.45377
S2 total	0.01949	0.04570	0.66983	0.71448
G0 total	0.01454	0.03687	0.69331	0.71567

**Table 2:** Changes in plasma N-glycome composition in the second time period (T2–T3) after meta-analysis. Seven glycan traits showed statistically significant changes in their levels after 6 months on different weight-maintenance diets (adjusted *p*-value < 0.05). AF—total glycans with antennary fucose; LB—total low branching glycans; HB—total high branching glycans; S0—total neutral glycans; S1—total monosialylated glycans; S2—total disialylated glycans; S3—total trisialylated glycans; S4—total tetrasialylated glycans; G0—total agalactosylated glycans; G1—total monogalactosylated glycans; G2—total digalactosylated glycans; G3—total trigalactosylated glycans; G4—total tetragalactosylated glycans; HM—total high-mannose glycans; B—total glycans with bisecting GlcNAc; CF—total glycans with core fucose.

	T2–T3			
Glycan	Effect	Standard Error	*p* Value	Adjusted *p* Value
AF total	−0.45084	0.04737	1.79 × 10^−21^	1.905 × 10^−20^
G4 total	−0.16539	0.05396	0.00218	0.00580
S4 total	−0.16587	0.05526	0.00269	0.00661
G3 total	0.17495	0.06677	0.00878	0.01873
G2 total	−0.18253	0.07246	0.01176	0.02353
LB total	−0.11801	0.04941	0.01693	0.03186
HM total	0.23105	0.10521	0.02809	0.04994
S1 total	0.07768	0.03809	0.04144	0.06630
HB total	0.09861	0.05152	0.05560	0.08472
S3 total	0.08903	0.06291	0.15702	0.21847
S2 total	−0.15549	0.11186	0.16449	0.21932
S0 total	0.08276	0.10077	0.41152	0.50649
G1 total	0.07344	0.09667	0.44741	0.51133
G0 total	0.06073	0.07868	0.44021	0.51133
B total	0.05390	0.07715	0.48477	0.53492
CF total	0.01978	0.09589	0.83656	0.83656

## Data Availability

The data presented in this study are available on request from the corresponding author. The data are not publicly available due to data being confidential records.

## Supplementary Materials

The following supporting information can be downloaded at: https://www.mdpi.com/article/10.3390/ijms232415772/s1. Supplementary Figure S1. Representative HILIC-UPLC-FLR chromatographic profile of the plasma protein 2-aminobenzamide-labeled N-glycome. Graphic representation of the glycan structures corresponding to each glycan peak (GP). In the case of multiple structures per GP, the upper structure is the major one, and the lower one is minor in abundance. EU: emission units. Supplementary Figure S2. Plasma N-glycome composition changes between different centers of the Diogenes study normalized to the first point. T1—time point 1; T2—time point 2; T3—time point 3; LB—total low branching glycans; HB—total high branching glycans; S0—total neutral glycans; S1—total monosialylated glycans; S2—total disialylated glycans; S3—total trisialylated glycans; S4—total tetrasialylated glycans; G0—total agalactosylated glycans; G1—total monogalactosylated glycans; G2—total digalactosylated glycans; G3—total trigalactosylated glycans; G4—total tetragalactosylated glycans; HM—total high-mannose glycans; B—total glycans with bisecting GlcNAc; CF—total glycans with core fucose; AF—total glycans with antennary fucose. Supplementary Figure S3. Plasma N-glycome composition changes between different diets normalized to the first point. GI—glycaemic index; T1—time point 1; T2—time point 2; T3—time point 3; LB—total low branching glycans; HB—total high branching glycans; S0—total neutral glycans; S1—total monosialylated glycans; S2—total disialylated glycans; S3—total trisialylated glycans; S4—total tetrasialylated glycans; G0—total agalactosylated glycans; G1—total monogalactosylated glycans; G2—total digalactosylated glycans; G3—total trigalactosylated glycans; G4—total tetragalactosylated glycans; HM—total high-mannose glycans; B—total glycans with bisecting GlcNAc; CF—total glycans with core fucose; AF—total glycans with antennary fucose.

## Abbreviations

BMI—body mass index; CVD—cardiovascular disease; T2D—type 2 diabetes; LCD—low-calorie diet; LP—low protein; HP—high protein; LGI—low glycemic index; HGI—high glycemic index; U(H)PLC—ultra-(high)-performance liquid chromatography; APPs—acute phase proteins; CRP—C-reactive protein; AGP—α1-acid glycoprotein; HPT—haptoglobin; A1AT—α1-antitrypsin; AACT—α1-antichymotrypsin; ApoB—the apolipoprotein B-100; GP—glycan peak.
