# Supplementary material for: Susceptibility of Human Plasma N-glycome to Low-Calorie and Different Weight-Maintenance Diets
Source: Int J Mol Sci. 2022 Dec 12;23(24):15772. doi: 10.3390/ijms232415772 (PMC9779867; doi:10.3390/ijms232415772)
Supplement: Supplementary file 1 [file ijms-23-15772-s001.zip › ijms-2005939-supplementary.pdf]

Supplementary files

Susceptibility of human plasma N-glycome to low-calorie and different weight-maintenance diets

Helena Deriš [a], Petra Tominac [a], Frano Vučković [a], Arne Astrup [b], Ellen E. Blaak [c], Gordan Lauc [a, d], Ivan Gudelj [a, e,]

[a] Genos Glycoscience Research Laboratory, 10000 Zagreb, Croatia

[b] University of Copenhagen, Department of Nutrition, Exercise and Sports, DK 2200 Copenhagen, Denmark

[c] Dept of Human Biology, NUTRIM, School for Nutrition and Translational Research in Metabolism, Maastricht University, 6200 MD Maastricht, Netherlands

[d] University of Zagreb, Faculty of Pharmacy and Biochemistry, 10000 Zagreb, Croatia

[e] University of Rijeka, Department of Biotechnology, 51000 Rijeka, Croatia

* Corresponding author, e-mail address: ivan.gudelj@uniri.hr (I. Gudelj).

Supplementary figures

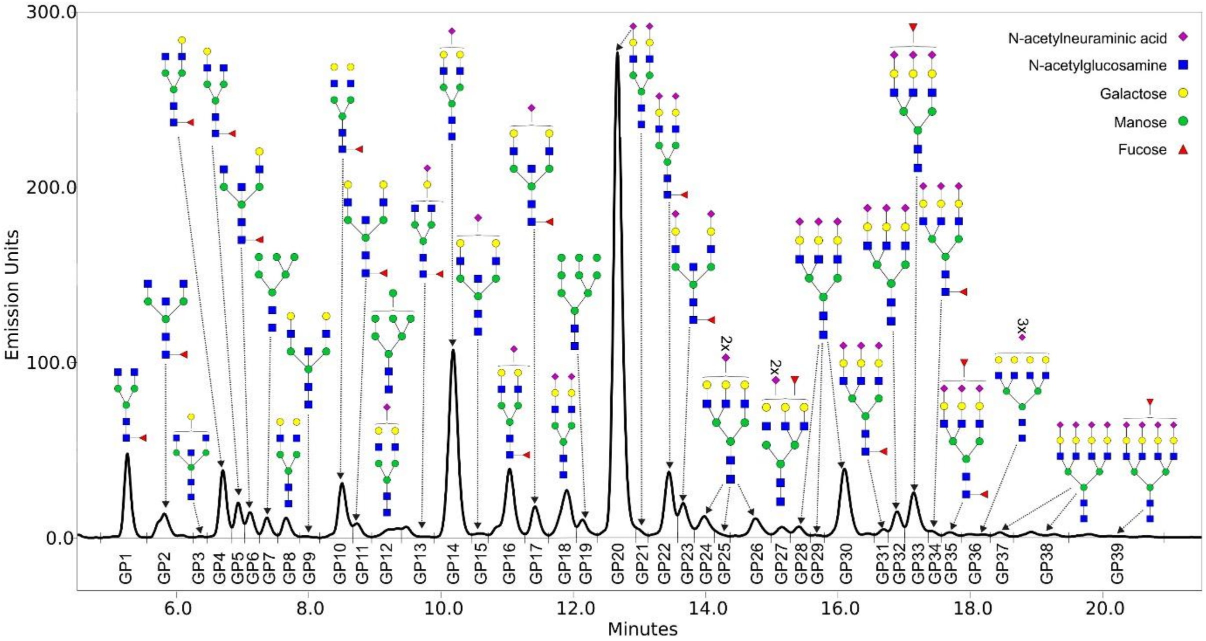

Figure S1: representative HILIC-UPLC-FLR chromatographic profile of the human plasma 2-AB labeled N-glycome. Graphic representation of the glycan structures corresponding to each of the 39 glycan peaks (GP) are shown. In the case of multiple structures per glycan peak, the upper structure is the major one, and the lower one is minor in abundance.

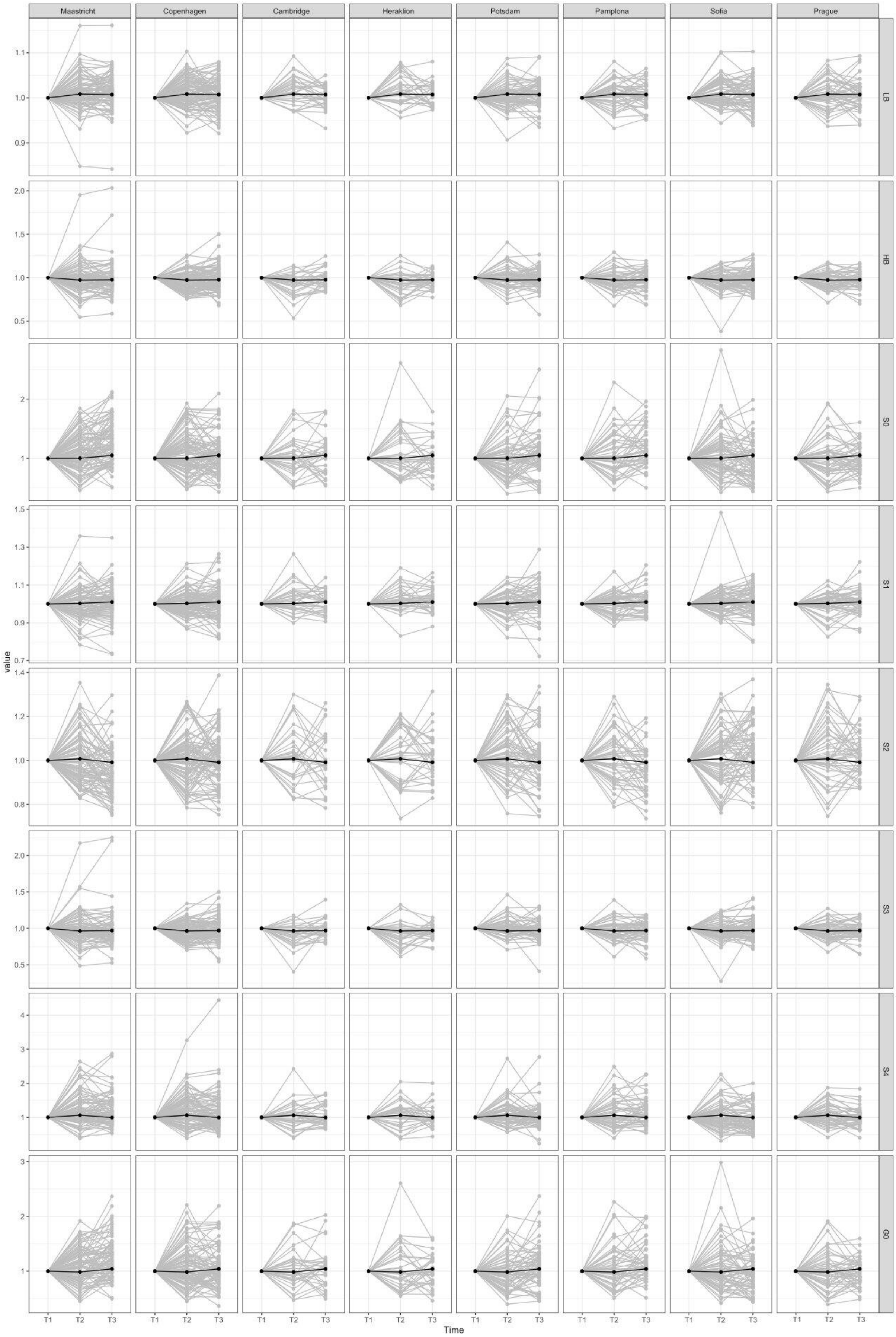
Maastricht
Copenhagen
Cambridge
Heraklion
Potsdam
Pamplona
Sofia
Prague
LB
HB
S0
S1
S2
S3
S4
G0
value
Time
T1
T2
T3

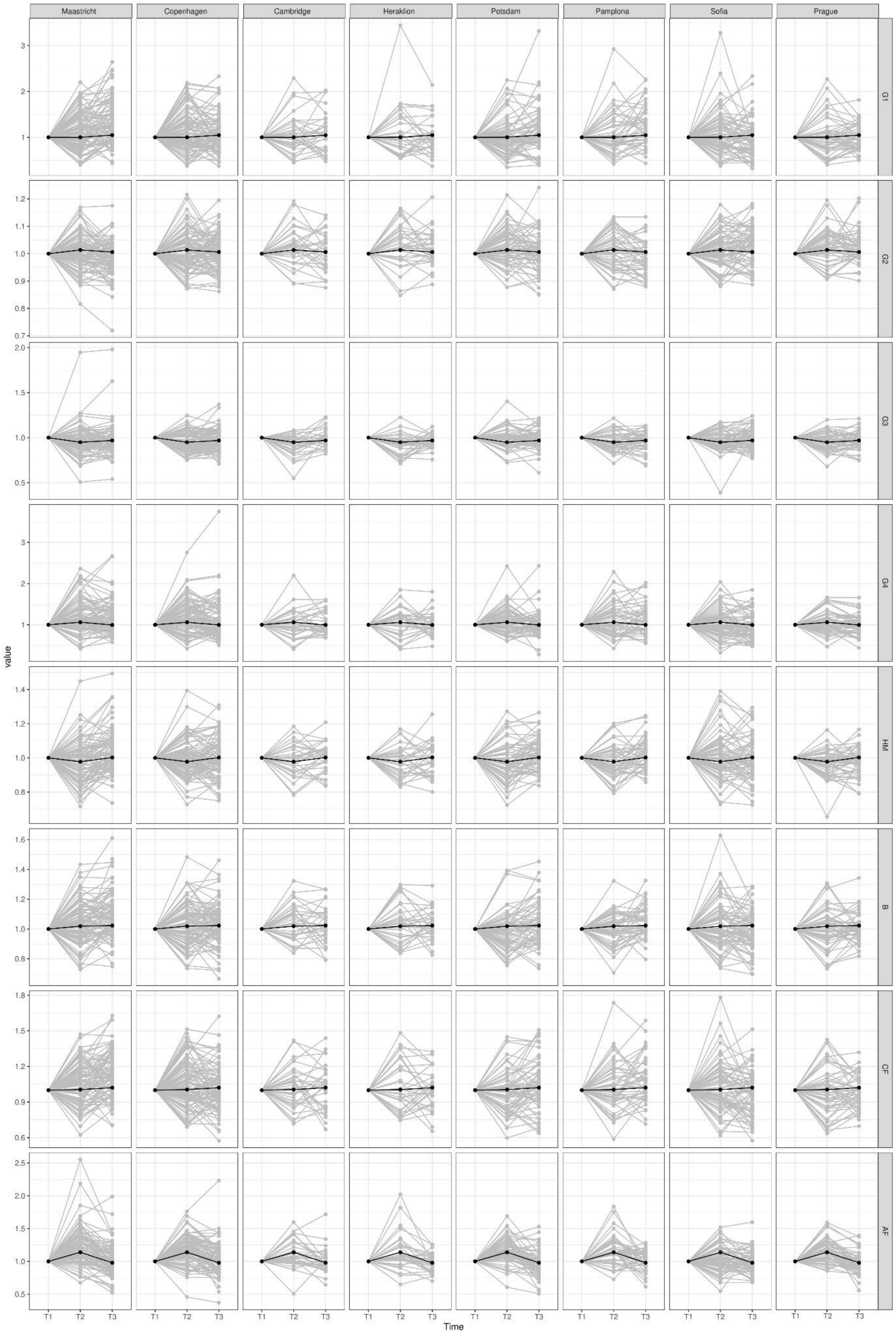

Maastricht
Copenhagen
Cambridge
Heraklion
Potsdam
Pamplona
Sofia
Prague
G1
G2
G3
G4
HM
B
CF
AF
value
Time
T1
T2
T3

Figure S2: plasma N-glycome composition changes between different centers of the Diogenes studies. N-glycome changes are normalized to the first point. T1 - time point 1, T2 - time point 2, T3 - time point 3, LB – total low branching glycans, HB – total high branching glycans, S0 – total neutral glycans, S1 – total monosialylated glycans, S2 – total disialylated glycans, S3 – total trisialylated glycans, S4 – toal tetrasialylated glycans, G0 – total agalactosylated glycans, G1 – total monogalactosylated glycans, G2 – total digalactosylated glycans, G3 – total trigalactosylated glycans, G4 – total tetragalactosylated glycans, HM – total high mannose glycans, B – total glycans with bisecting GlcNAc, CF – total glycans with core fucose, AF – total glycans with antennary fucose.

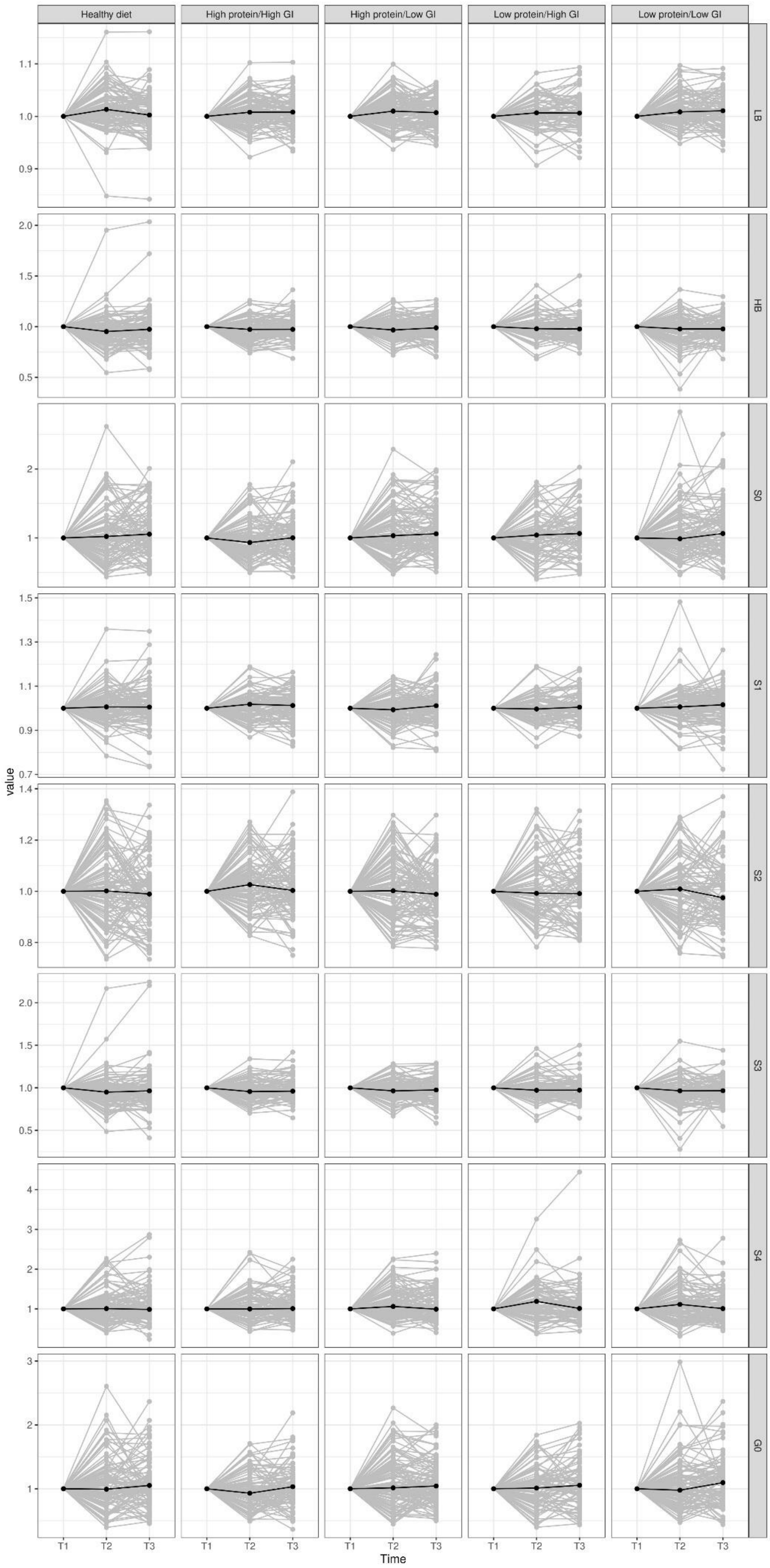
Healthy diet
High protein/High GI
High protein/Low GI
Low protein/High GI
Low protein/Low GI
LB
HB
S0
S1
S2
S3
S4
G0
value
Time
T1
T2
T3

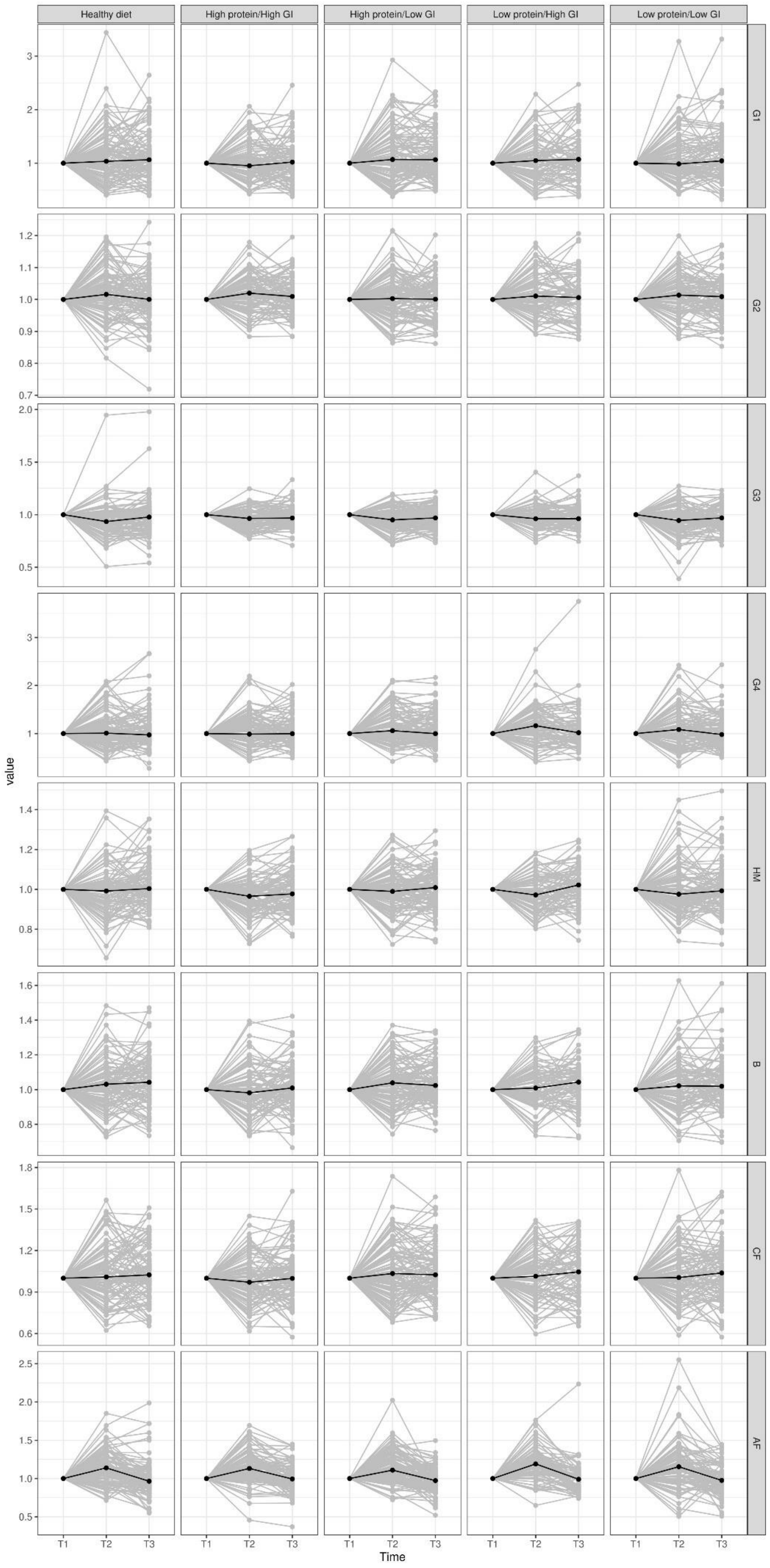
Healthy diet
High protein/High GI
High protein/Low GI
Low protein/High GI
Low protein/Low GI
G1
G2
G3
G4
HM
B
CF
AF
value
Time
T1
T2
T3

Figure S3: plasma N-glycome composition changes between different types of weight-maintenance diets. Obtained changes are normalized to the first point. GI – glycaemic index, T1 - time point 1, T2 - time point 2, T3 - time point 3, , LB – total low branching glycans, HB – total high branching glycans, S0 – total neutral glycans, S1 – total monosialylated glycans, S2 – total disialylated glycans, S3 – total trisialylated glycans, S4 – toal tetrasialylated glycans, G0 – total agalactosylated glycans, G1 – total monogalactosylated glycans, G2 – total digalactosylated glycans, G3 – total trigalactosylated glycans, G4 – total tetragalactosylated glycans, HM – total high mannose glycans, B – total glycans with bisecting GlcNAc, CF – total glycans with core fucose, AF – total glycans with antennary fucose.
